# Ethical Guidance for Medical Schools and Medical Students in High-Income Countries for Clinical Electives in Low- and Middle-Income Countries: A Systematic Review

**DOI:** 10.5334/pme.2150

**Published:** 2026-07-14

**Authors:** Judith van de Kamp, Lara Alessandra de Luca, Cato Elisabeth Andree Corsten, Joyce Linda Browne, Ross Upshur, Hannah Brown Amoakoh, Johannes van Delden, Rieke van der Graaf

**Affiliations:** 1 Department of Global Public Health and Bioethics, University Medical Center Utrecht, the Netherlands; 2 University Medical Center Utrecht, the Netherlands; 3 Erasmus University Medical Center Rotterdam, the Netherlands; 4 University of Toronto, Canada; 5 University of Ghana, Ghana; 6 Department of Global Health, University Medical Center Utrecht, the Netherlands

## Abstract

**Introduction:**

Academic interest in ethical guidance for medical students from High-Income Countries (HICs) undertaking clinical electives in Low- and Middle-Income Countries (LMICs) has increased. Earlier ethical frameworks laid important groundwork but predate contemporary debates on social justice in global health. This study reviews recent literature to assess how these debates shape and produce ethical guidance, distinguish between ethical student conduct and elective programs, and how such guidance is translated into practice.

**Methods:**

A systematic review was conducted on ethical guidance for HIC medical schools and students regarding LMIC electives. Articles published between January 2018 and October 2023 were identified from PubMed, Embase, and Global Health. Thematic synthesis was combined with analysis of authorship (pose) and intended audience (gaze).

**Results:**

Of 711 articles, 24 met inclusion criteria. Five interconnecting themes emerged: humility, reflection, local embeddedness, equitable partnerships, and student health and well-being. While these align with existing frameworks, guidance remains largely conceptual, with limited attention to implementation and accountability across ethical student conduct and program design, with responsibility predominantly located at the level of HIC students. Pose and gaze analysis revealed epistemic imbalances in authorship and intended audiences. Most articles reflected HIC-based perspectives, with North American authors overrepresented and no solely LMIC-authored articles.

**Discussion:**

Ethical guidance remains dominated by HIC perspectives and focused on student conduct rather than program structures. By distinguishing between individual and institutional ethics, this study highlights the need for actionable, co-created frameworks that address accountability, equity, and sustainability in LMIC electives, including attention to institutional responsibility and LMIC perspectives.

## Introduction

Since the early nineties, it has become increasingly popular for medical students in universities in High-Income Countries (HICs) to enroll in a clinical elective in a Low- or Middle-Income Country (LMIC) [1,2]. HIC universities have embraced LMIC clinical electives as part of their medical curricula to provide students the opportunity to broaden their horizon for personal and professional development, through the anticipated improvement of intercultural competences, language skills, and knowledge on health systems and tropical diseases with different clinical exposure [1,2,3,4,5,6].

Various studies from the past fifteen years show that ethical guidance is essential for HIC students and institutions that offer LMIC-based clinical electives [2,3,6,7,8,9,10]. Ethical considerations for students include the risk of practicing outside scope of training (POST) and thereby jeopardizing patient safety [5,11]. POST can arise in a context of local health workforce shortages because of a high demand of clinical activities outside the students’ scope of training and too little time for supervision [6,9]. Additionally, POST can be caused by a lack of clarity on the part of local supervisors on the students’ level of training, as medical curricula and student responsibilities linked to training vary widely across countries [12,13,14]. As a consequence, a local supervisor might ask a HIC medical student to carry out a procedure based on the assumption that the student is familiar with the procedure, while the student might not yet have sufficiently practiced this procedure as part of their home institution’s medical training program. Finally, there are other risks related to the HIC students being outsiders to the specific cultural and socio-economic and low-resource context, which affects their understanding of how these contextual factors affect the practice of medicine [2,11,12,15,16,17]. The limited contextual understanding of the medical setting may hinder the building of relationships with local health workers and students [12,18] and, again, jeopardize adequate provision of care and patient safety [9].

Amidst these known challenges and in the absence of concrete guidance, it has long remained unclear to HIC medical schools how to best prepare students for international medical electives taking place in LMICs – including how to support students in navigating challenges and dilemmas they could face. In 2009, as a starting point for the development of an applicable framework for global health ethics for students, Pinto and Upshur presented four actionable principles, or virtues to guide student conduct: humility, introspection, solidarity, and social justice [19]. Over the years, other ethical frameworks appeared in the academic body of literature, such as the WEIGHT Working Group guidelines launched in 2010 [20] and the ethical principles by Melby and colleagues (2016) [21]. In a systematic review carried out by Rahim and colleagues, 17 articles were identified on ethical guidance for LMIC clinical electives (2016) [22]. What characterized all publications thus far, is that these were published solely by authors affiliated with institutions in the United States and Canada [23].

Within recent years, critical thinking on global power dynamics, equity and justice, and decolonizing global health gained ground in academic debates and discourse [[Bibr B24][Bibr B25][Bibr B26][Bibr B27][Bibr B28][Bibr B29][Bibr B30][Bibr B31][Bibr B32]]. Together with environmental sustainability concerns, this thinking has led to calls to move away from short-term unidirectional international engagement to more sustainable and equitable North-South educational and research partnerships [3,33,34,35,36]. Yet, despite this recent discourse, it remains unclear to what extent these debates have informed or are reflected in ethical guidance on clinical electives in LMICs. Therefore, this systematic review aims not only to analyze recent literature on ethical principles for medical schools and students in HICs undertaking clinical electives in LMICs, but also to explore how this guidance is produced in terms of authorship and audience.

## Methods

We conducted a systematic review informed by the RESERVE guidelines for systematic review reporting in ethics [37]. Although the review has characteristics that overlap with scoping reviews, we retain the term systematic review because the review process was systematically conducted, including structured database searches, dual independent screening, explicit inclusion and exclusion criteria, and transparent data extraction procedures. In addition, the purpose of this review was not only to map the literature but also to systematically synthesize normative ethical guidance within LMIC clinical electives. This positioning aligns with the RESERVE framework, which supports systematic reviews of normative and ethics literature despite methodological heterogeneity. The eligibility criteria are listed in Table 1.

**Table 1:** Inclusion and Exclusion Criteria, From January 1 in 2018 Until October 18 in 2023.


	INCLUSION CRITERIA	EXCLUSION CRITERIA

**Population**	– Medical students enrolled in a formal medical program at a HIC academic institution.	– Medical students not in a formal medical training program, and/or; – Other undergraduate students or unspecified undergraduate trainees, and/or; – Residents, junior doctors, or unspecified graduate trainees, and/or; – Qualified health workers and volunteers.

**Exposure**	– Elements of a clinical elective program in a LMIC (World Bank, 2023), and; – Elective conducted independently, outside a broader HIC medical mission (e.g., surgical mission trip).	– Elements of a clinical elective in a HIC (World Bank, 2023); – Elective conducted within a broader HIC medical mission (e.g., surgical mission trip).

**Outcome**	– Suggestions, preconditions, or requirements for an ethically justified program, including a) virtues and skills for HIC students on LMIC electives, b) individual, institutional, and educational aspects of ethically responsible electives, and; – Suggestions for various student groups (e.g. nursing), either specific to each group or applicable to all.	– Not related to suggestions, preconditions, or requirements of an ethically justified program; – Suggestions for various student groups (e.g. nursing) without differentiation, making it unclear how they apply specifically to medical students.

**Type of papers**		Conference abstracts

**Language**	English	All other languages


Abbreviations: HIC: High-Income Country. LMIC: Low- or Middle-Income Country.

### Search strategy

Four authors (CC, LL, JK, RG) contributed to the development of a search strategy supported by a university librarian. The search terms were also reviewed and refined by the author team, who have expertise in global health, medical education, and the ethics of medical electives, to ensure their relevance and comprehensiveness. The search was conducted on October 18, 2023, using PubMed, Embase, and Global Health. A separate search string was constructed for each database based on four elements: 1) medical students, 2) international electives, 3) ethics, and 4) LMIC, including relevant synonyms. The search strings appear in the Supplementary Material.

We limited our search to literature published from 2018 onward. This decision builds on the systematic review by Rahim et al. (2016), which covered ethical guidance for LMIC clinical electives up to approximately 2016 [22]. The period 2018–2023 was therefore selected as a conceptual window to capture developments in ethical guidance in the context of increasing academic attention to equity, decolonization, and sustainability in global health education.

### Selection process

Two authors (LL and JK) independently screened titles and abstracts for relevance using the Rayyan web tool [38]. Any disagreements were discussed until consensus was reached. Full-text articles were independently assessed for eligibility by the same two authors based on predefined inclusion and exclusion criteria, with discrepancies resolved through discussion and consensus. Given the wide variability in terminology for LMIC clinical electives (see Results), we documented all synonyms encountered and ensured consistent coding by mapping them to a single category (‘LMIC clinical electives’) during data extraction and thematic synthesis. This approach allowed us to synthesize findings across studies despite inconsistent terminology. Following RESERVE guidelines, all identified articles are in bold in the reference list.

### Data extraction

Data extraction was performed by two authors (LL and JK) using standardized spreadsheets. The first tracked study characteristics: title, first and last author affiliation, paper type, scope and aim. The second captured ethics-related information on LMIC clinical electives, including principles and virtues for HIC students, as well as individual, institutional, and educational elements for ethical electives. Textual data was documented as citations.

### Data synthesis, including identification of codes and themes

A thematic synthesis identified ethics-related themes in LMIC clinical electives. The coding process was inductive, starting from the data and generating an initial set of 72 preliminary codes. Pinto and Upshur’s framework (2009) [19] was not applied deductively from the outset, but was used as a sensitizing and interpretive lens in later stages of the analysis.

One author (LL) conducted the initial coding, after which the 72 codes were iteratively discussed within the core team (JK and RG). Closely related codes were grouped into 11 higher-order codes based on conceptual similarity and relevance to ethical guidance, focusing on the ‘what,’ ‘why,’ and ‘who’ of LMIC electives to capture key ethical aspects, underlying rationales, and stakeholder roles. Through iterative discussions, brainstorming sessions, and visual mapping using flipcharts, these 11 codes were further consolidated into five overarching themes. Saturation was reached when additional consolidation did not yield new themes or meaningful distinctions. The themes emerged as the most representative of the underlying structures in the data, and this number (five) was determined based on saturation: adding more themes would not have provided substantially new insights but rather led to artificial fragmentation of the data.

Authorship was analyzed using Abimbola’s concepts of pose (author’s standpoint) and gaze (intended audience) [39], based on author affiliations and article content.

### Quality appraisal

Given the heterogeneity and predominantly normative nature of the included literature, no formal risk-of-bias or quality scoring tool was applied. Instead, we conducted a descriptive appraisal of the included publications based on study type, conceptual scope, and relevance to ethical guidance on LMIC electives (Table 2). This approach aligns with the RESERVE framework for systematic reviews of ethics literature, which prioritizes interpretive synthesis over hierarchical quality assessment.

**Table 2:** Characteristics of Included Articles.


NR	TITLE	AFFILIATION FIRST AUTHOR	AFFILIATION LAST AUTHOR	TYPE OF PAPER	SCOPE OF PAPER	AIM OF PAPER	LMIC DESTINATIONS	POSE	GAZE

**1**	Ten Questions to Guide Learners Seeking Equitable Global Health Experiences Abroad (2023)	Medical student at University of Michigan, USA	University of Global Health Equity, Butaro, Rwanda	Scholarly Perspective	Equitable global health experiences	To offer 10 guiding questions to support students in choosing equitable LMIC electives, for mutually beneficial partnerships	Not specified	USA/Rwanda	HIC students and HIC institutions

**2**	International Health Electives: defining learning outcomes for a unique experience (2023)	Department of Obstetrics and Gynaecology, University Medical Center Groningen	University Medical Center Groningen	Research	Learning outcomes for International Health Electives (IHEs)	To clarify the possible learning outcomes for International Health Electives (IHEs) in LMICs as perceived by HIC students to facilitate incorporation of IHEs in GHE and help clarify their link to professional performance.	Ghana, Malawi, Nepal, Rwanda Uganda, Sierra Leone, Suriname, and South Africa	Netherlands/Netherlands	HIC institutions

**3**	From the Global North to the Global South: preparing students for away rotations (2023)	Uni of Catanzaro, Italy	Uni of Catanzaro, Italy	Commentary	LMIC electives by HIC med students	To comment on students’ preparation for away rotations based on the authors’ experiences and opinions alongside a review of papers	Not specified	Italy/Italy	Unspecified

**4**	Ethical Considerations of Climate Justice and International Air Travel in Short-Term Electives in Global Health (2023)	Uni of Minnesota, USA	Division of Hospital Medicine, University of Minnesota, USA	Perspective	Climate change considerations in STEGH programs	To suggest several strategies that HIC GH medical education programs could implement to act on climate change health impacts and consider decolonization and decarbonization	Not specified	USA/USA	HIC institutions

**5**	Managing the Unpredictable: Recommendations to Improve Trainee Safety During Global Health Away Electives (2022)	Cape Western Reserve University, Ohio, USA	Yale School of Medicine, New Haven, Connecticut, USA	Original research	Student safety during GH away rotations	To provide critical safety considerations requiring additional guidance for programs and students on LMIC clinical electives	Not specified	USA/USA	HIC students and HIC institutions

**6**	Medical students from German-speaking countries on abroad electives in Africa: destinations, motivations, trends and ethical dilemmas (2022)	University of Freiburg, Germany	Department of emergency Medicine, Cantonal Hospital of Neuchatel, Neuchatel, Switzerland	Research	Motivations of HIC medical students on LMIC clinical electives	To provide insights into factors driving medical student to travel to Africa for clinical electives, as an opportunity for HIC med schools to restructure their international elective programs	Benin, Cameroon Ethiopia, Gambia Ghana, Ivory Coast Kenya, Malawi Morocco, Namibia Botswana, Mauritius, Senegal, Seychelles, Rwanda, South Africa, Tanzania, Togo, Tunisia, Uganda, and Zambia	Germany/Switzerland	HIC institutions

**7**	Low- and Middle-Income Country Host Perceptions of Short-Term Experiences in Global Health: A Systematic Review (2021)	Perelman School of Medicine, Philadelphia, USA	Yale School of Medicine, Yale University, New Haven, Connecticut, USA	Review	LMIC host perceptions of STEGH	To summarize LMIC host perceptions of STEGH, develop best practices for STEGH that provide guidance for HIC and LMIC stakeholders	Kenya, the Philippines, Namibia, Angola China, Ethiopia Ghana, Guyana India, Nepal Rwanda, South Africa, Uganda Vietnam, Argentina Peru, Malawi, Tanzania, Zambia Botswana, Eswatini Bolivia, and Benin	USA/USA	HIC and LMIC institutions

**8**	Embedding international medical student electives within a 30-year partnership: the Ghana-Michigan collaboration (2020)	University of Michigan, USA	University of Michigan, USA.	Research article	The Ghana-Michigan collaboration	To explore the University of Michigan med student elective in Ghana within the context of the Ghana-Michigan collaborative	Ghana	USA/USA	Unspecified

**9**	Developing Ethical and Sustainable Global Health Educational Exchanges for Clinical Trainees: Implementation and Lessons Learned from the 30-Year Academic Model Providing Access to Healthcare (AMPATH) Partnership (2020)	Indiana University School of medicine, Indiana, USA AND Moi University Medical School Eldoret, Kenya	College of Health Sciences, Moi University, Eldoret, Kenya	Perspective	Lessons learned from the AMPATH partnership for educational exchanges for clinical trainees	To describe the bilateral exchange of trainees in the AMPATH partnership of 12 universities in North America and 1 in Kenya, and hereby provide a model for developing institutional HIC-LMIC partnership	Kenya	USA/Kenya	HIC and LMIC institutions

**10**	Perspectives and Solutions from Clinical Trainees and Mentors Regarding Ethical Challenges During Global Health Experiences (2020)	Harvard Med school, USA	Harvard Medical School, USA	Original research	Staff and students’ perspectives on ethically responsible STEGH	To describe perspectives and potential solutions in STEGH as identified by HIC students and staff, to help prevent, mitigate or resolve ethical challenges.	Not specified	USA/USA	HIC students and HIC institutions

**11**	Rethinking Goals: Transforming Short-Term Global Health Experiences Into Engagements (2020)	University of Arkansas for Medical Sciences, Arkansas, USA	University of Texas Medical Branch, Texas, USA	Perspective	Transformative learning goals for STEGH, reconceptualizing STEGH as engagements	To present and encourage the use of 5 key learning goals for STEGH participants for transformative learning	Not specified	USA/USA	HIC institutions

**12**	Structured medical electives: a concept whose time has come? 2019	Kings College London, UK	Medical School for International Health, Ben-Gurion University of the Negev, Beer-Sheva, Israel	Review	Roles for HIC medical schools organizing international clinical electives	To provide insights into the role of HIC med schools in organizing international clinical electives	Ghana, Ethiopia India, Nepal Sri Lanka, Peru, Malawi, and Zambia	UK/Israel	HIC institutions (UK medical schools primarily, and also HIC medical schools in general)

**13**	Extent, nature and consequences of performing outside scope of training in global health. 2019	Northwestern University, Chicago, USA	Massachusetts General Hospital, USA AND Harvard Medical School, Boston, USA.	Research	Students performing outside scope of training	To explore the frequency, nature, circumstances and consequences of performing outside scope of training (POST)	Not specified	USA/USA	Unspecified

**14**	Are you ready? A systematic review of pre-departure resources for global health electives (2019)	John Hopkins Center for GH, Baltimore, USA AND John Hopkins Bloomberg School of Public Health, Baltimore, USA	Johns Hopkins Center for Global Health, Baltimore, USA AND Johns Hopkins School of Medicine, Baltimore, USA	Review	PDT for LMIC clinical electives	To quantify the plethora of PDT for LMIC electives	Not specified	USA/USA	HIC institutions in collaboration with LMIC sites

**15**	Development of a Novel Global Surgery Course for Medical Schools (2019)	Massachussets Hospital and Havrard Med School, USA	Lund University, Sweden	Review	A multinational exchange course in global surgery	to share evaluation results from a multinational exchange course in global surgery, involving students from Sweden, USA and Zimbabwe (with clinical elective for the Swedish and American in Harare, and clinical elective in Sweden for the Zimbabwian)	Zimbabwe	USA/Sweden	HIC institutions

**16**	Global health electives: Ethical engagement in building global health capacity (2020)	University of Calgary, Alberta, Canada	University of Calgary, Alberta, Canada	Research	The impact of Canada clinical electives on host institutions in Tanzania and Uganda	To provide host perspectives to help better design ethically responsible LMIC electives	Tanzania and Uganda	Canada/Canada	HIC institutions

**17**	Reciprocity? International Preceptors’ Perceptions of Global Health Elective Learners at African Sites (2019)	University of Utah, USA	Baylor College of Medicine, USA	Original research	Benefits and burdens of LMIC clinical electives from host perspectives	To provide insights in LMIC host preceptors’ perceptions regarding benefits and burdens of hosting STLs	Malawi and Lesotho	USA/USA	Unspecified (other than a ‘need for institutions to identify mutuality’)

**18**	Longitudinal Service Learning in Medical Education: An Ethical Analysis of the Five-Year Alternative Curriculum at Stritch School of Medicine (2018)	University of Illinois, Chicago, USA	none	Perspective	The GH Fieldwork Fellowships in Bolivia and its benefits for students	To describe the one-year GH Fieldwork Fellowship (GHFF) in Bolivia as a curricular model to foster professionalism and social values	Bolivia	USA	Unspecified

**19**	The ethical experiences of trainees on short-term international trips: a systematic qualitative synthesis (2018)	John Hophins Hospital, Baltimore, USA	John Hopkins University, Baltimore, USA	Review	STINTTs ethical experiences	To identify ethical issues medical trainees encounter during STINTTs	Not specified	USA/USA	HIC institutions

**20**	Pre-departure Training for Healthcare Students Going Abroad: Impact on Preparedness (2018)	John Hopkins School of Med, Baltimore, USA	Department of Gynecology and Obstetrics, Johns Hopkins School of Medicine, Baltimore, MD, USA	Original research	The efficacy of PDT in preparing students for LMIC clinical electives	To identify characteristics of PDT associated with participants’ reporting levels of preparedness.	Not specified	USA/USA	HIC institutions

**21**	International medical electives in selected African countries: a phenomenological study on host experience (2018)	University of Notre Dame, Australia	University of Notre Dame, Australia	Research	Host perspectives on IMEs	To explore the host experience on international medical electives as a selection of hospitals in LMICs in Africa, to inform toe preparation of GH curricula.	Uganda, South Africa, and Eswatini	Australia/Australia	HIC institutions

**22**	Ethical reflection for medical electives (2018)	University of Birmingham Medical School, UK	University of Birmingham Medical School, UK	Original research	Ethical considerations for LMIC medical electives	To identify ethical considerations for elective students, based on interviews with UK students in the Solomon Islands	Solomon Islands	UK/UK	HIC institutions, and specifically primarily UK institutions

**23**	A Comparison of the Expectations and Experiences of Medical Students From High-, Middle-, and Low-Income Countries Participating in Global Health Clinical Electives (2018)	Boston USA	Yale University USA	Research	LMIC and HIC student perspectives on GH clinical electives	Identifying differences and similarities in the experiences of HIC students on LMIC clinical electives and LMIC students on HIC clinical electives,	“six in Latin America, three in Africa, three, in Asia, and two in the Caribbean”	USA/USA	Unspecified

**24**	Ethical dilemmas during international clinical rotations in global health settings: Findings from a training and debriefing program (2018)	San Francisco Med center, USA	yale University, USA	Research article	An ethics workshop to prepare US students for an international clinical elective	To describe the impact of an ethics workshop in the context of international clinical rotations to inform future interventions	China, South Africa, Uganda, Argentina, Indonesia, Thailand, Peru, Jamaica, and Dominican Republic	USA/USA	HIC institutions


### Authorship reflexivity

This review originated as a local educational project at UMC Utrecht, aimed at training master’s students in systematic review methodology under faculty supervision. At that stage, the project was not conceived as an international collaboration, but rather as an internal exercise: to examine the ethical guidance currently available in the literature to better prepare our students for international medical electives and to strengthen institutional support and supervision.

When the synthesis revealed broader relevance for global health education, we developed the work into a manuscript and subsequently invited additional colleagues with complementary expertise, also from our partner institution in Ghana. Although our author team reflects existing global imbalances, this is not the same as uncritically reproducing them. We deliberately avoided tokenistic authorship practices, such as inviting LMIC without meaningful involvement, as we consider that ethically inappropriate.

Finally, UMC Utrecht has long-standing equitable collaborations with partner hospitals in Ghana, Malawi, and Suriname, with whom we co-develop educational programs and research. These partnerships will be central to the next phase of work, which aims to co-create guidance that incorporates the perspectives and priorities of all partner institutions involved in (reciprocal) international student exchanges.

## Results

### Publication selection process

**Figure 1:**
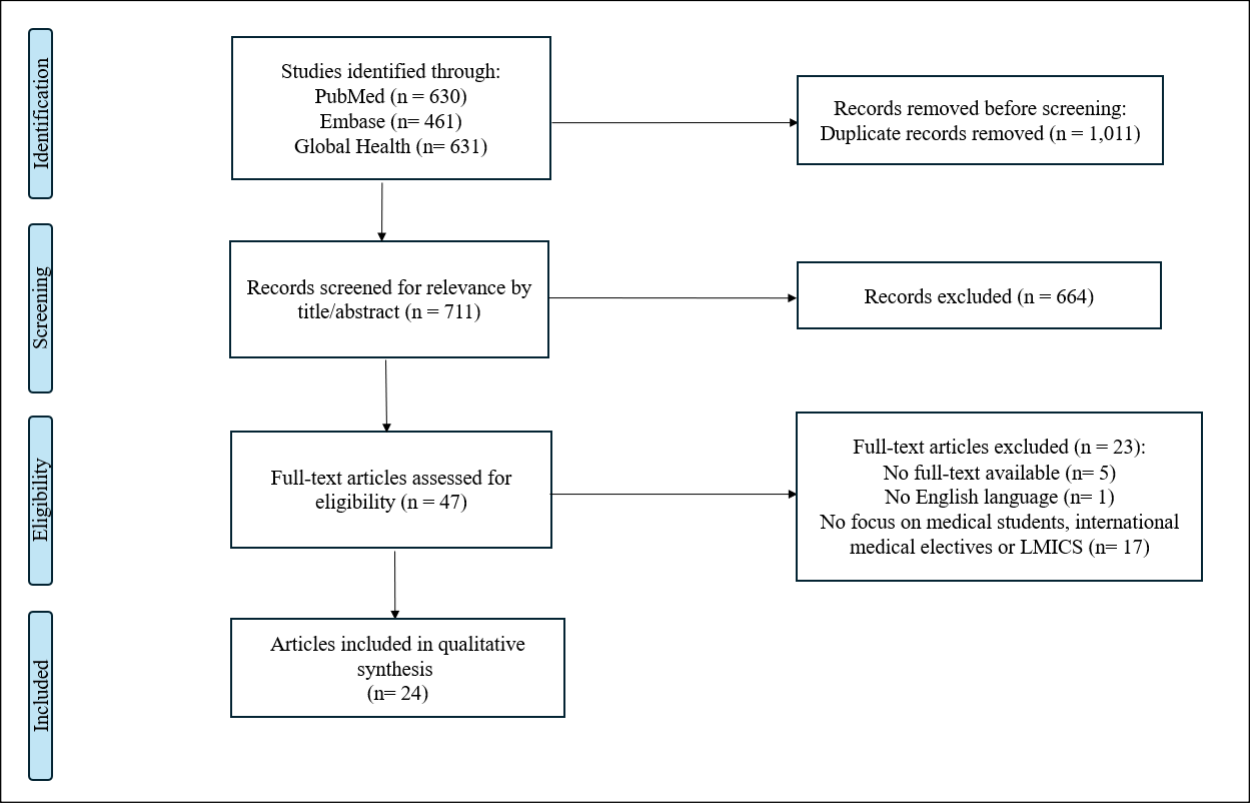
Flowchart of the Literature Study Search and Inclusion Process.

The search identified 711 unique publications, 47 of which were screened full text, and 24 were included in the review after close-reading, as shown in Figure 1.

### Characteristics of publications

The majority of articles were original research studies (13), along with 5 perspectives, 1 commentary and 5 reviews. Table 2 presents the characteristics of included articles. Table 3 shows the categorization of articles’ pose based on first and last author’s affiliation, with 17 out of the 24 articles (71%) from the United States. There were no articles with first and last authorship solely from LMIC institutions. We also assessed the articles’ gaze and found that in 67% (N = 16) of the articles, authors were explicitly writing for a HIC audience, in 12,5% (N = 3) articles explicitly for both HIC and LMIC audiences. In 25% (N = 6) of the articles, authors had not specified their gaze, and none of the articles were explicitly written for an LMIC audience.

**Table 3:** Pose Based on First and Last Author’s Affiliation for Articles Included.


HICs		HIC AND LMIC	LMICs

AUTHORS FROM 1 HIC (n = 19)	AUTHORS FROM 2 HICs (n = 3)	AUTHORS FROM 1 HIC & 1 LMIC	AUTHORS FROM 2 LMICs

USA (14)	Germany-Switzerland (1)	USA-Rwanda (1)	None
Italy (1)	United Kingdom-Israel (1)	USA-Kenya (1)	
The Netherlands (1)	USA-Sweden (1)		
Canada (1)			
Australia (1)			
United Kingdom (1)			

**HICs:** **n = 22 (92%)**		**HIC and LMICs:** **n = 2 (8%)**	**LMICs:** **0%**


Abbreviations: HIC: High-Income Country. LMIC: Low- or Middle-Income Country. USA: United States of America.

As noted in the methods, there is no consistency in terminology within the international electives literature. We identified 16 synonyms for LMIC clinical electives, which we could confirm only after reading abstracts or full texts (global health electives; global health experiences; global health experiences abroad; global health away electives; global health away rotations; global health fieldwork fellowship; global health clinical electives; abroad electives; international health electives; international medical electives; international medical students electives; international clinical rotations; away rotations; short-term electives in global health, referred to as STEGH; short-term experiences in global health, also referred to as STEGH; short-term international trips, referred to as STINTTs). This variability in terminology complicates interpretation, comparison, and synthesis of findings across studies, highlighting the need for clearer, standardized definitions when reporting on LMIC clinical electives.

### Results of synthesis

**Figure 2:**
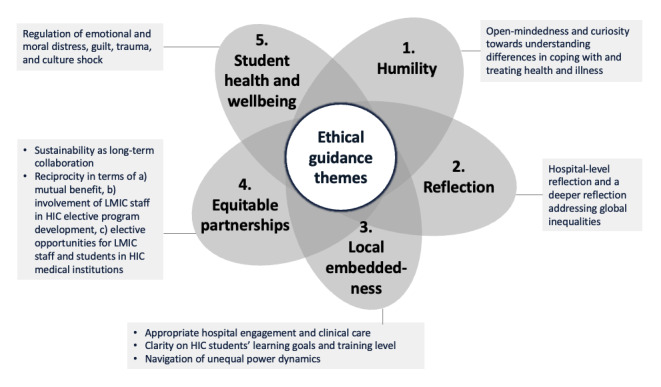
Interrelated Ethical Themes in LMIC Clinical Electives and Their Meaning Based on the Synthesis Results.

We identified five main ethical themes, most of which matched with Pinto and Uphur’s initial principles: 1) humility, 2) reflection, 3) local embeddedness, 4) equitable partnerships, and 5) student health and well-being. These themes, visualized in Figure 2, are conceptually interrelated and are described by their definition (‘what’), importance (‘why’), and the roles and responsibilities of involved people and institutions (‘who’). While these themes largely align with existing frameworks, their distribution across individual and institutional levels reveals how ethical responsibility is unevenly located across students and programs, challenging the dominant focus on individual student conduct in favour of a more structural understanding of ethics in LMIC electives.

### Theme 1: Humility

#### What

The significance of humility for HIC students during LMIC clinical electives is highlighted in 17 articles [40,41,42,43,44,45,46,47,48,49,50,51,52,53,54,55,56]. Humility, particularly cultural humility, is described as *“an interpersonal stance that is other-orientated rather than self-focused, characterized by respect and lack of superiority towards an individual’s cultural background and experience”* [43(p467)] and *“a lifelong practice of self-reflection and critique that involves entering relationships with the ability to adapt one’s own beliefs and values in consideration of the other”* [40(p1109)]. The emphasis on culture is explained by the differences in cultural norms between HIC students and patients, and staff and students in LMIC settings. These are inextricably linked to healthcare, as illustrated by this quote: *“[…] perhaps the preparation needed would be to help them (elective student) understand how they see their practice of medicine as having a cultural bias”* [53(p1431)].

The articles that discuss humility in broader terms emphasize open-mindedness and curiosity about different cultural norms and values on health and healthcare, religion [41], language [42], health literacy of the population [41], the conditions endemic to the host site [41,43], economics linked to the macro level of a health system and to the micro level of individual patients and how they cope with illness [41], and healthcare professional roles at the host site [43]. In one of the articles, there is reference to the term ‘global humility’ [47]. It is defined as *“the sense of awe and wonder at what we do not understand in relationship to health and illness combined with the acknowledgment that we have much to learn from host communities”* [47(p33)].

#### Why

It appears that students are encouraged to practice humility during their LMIC clinical electives to improve patient care and patient communication, and secondly to benefit staff relationships. It is best summarized by this quote: *“Ultimately, a focus on nurturing cultural humility […] may further diminish ethnocentric attitudes in both the clinical and interpersonal arenas and foster richer relationships between hosts and visitors”* [43(p467)].

A third reason to practice humility and maintain an open mind is its positive impact on the student’s future healthcare work upon return to their home country. HIC students reported significant improvements in their intercultural communication skills only after their return home [43].

#### Who

Although there is a clear role for the HIC students themselves to adopt and nurture the virtue of humility, most articles emphasize the responsibility of HIC medical schools to support students in doing so. There is one article that specifically calls upon students to take up this responsibility. This article encourages students to reflect on cultural humility by completing a reflection tool which they can do independently or with the guidance of a HIC faculty member who is familiar with the host setting. The majority of the articles call for Pre-Departure Training that should be organized by HIC medical institutions to help students adopt and nurture the virtue of humility.

Apart from *students* having to develop the virtue of humility supported by their sending institutions, there is one article that suggests international electives take place within formalized equitable partnerships, and within this context suggests “*to promote and role model cultural humility at all levels of the partnership (ranging from institutional to individual host–visitor interactions)”* [43(p466)].

#### Implementation and tensions

Although humility is consistently emphasized as a key attribute for students undertaking LMIC electives, its operationalization is largely limited to Pre-Departure Training and individual reflection tools, with little guidance on how it is reinforced in practice during electives. Moreover, responsibility is primarily placed on HIC students, with limited attention given to the role of HIC institutions in structuring, governing and modelling such humility within program design, and even less consideration of how host institutions engage with, shape, and evaluate these processes.

### Theme 2: Reflection

#### What

Most articles emphasize the need for HIC students to practice reflection during LMIC clinical electives. One type, hospital-level reflection, involves HIC students critically examining their role within the host hospital, aligning their motivations and learning goals, and considering their impact, such as potentially overburdening local staff [40].

The other type of reflection is a deeper reflection that addresses global inequalities shaping these electives. Terms capturing this approach are ‘critical reflection’ [42,45,46,50,57], ‘structural awareness’ [47], ‘contextual inquisitiveness’, and ‘critical engagement in the pursuit of creating equitable and just societies’ [47]. Structural awareness, described by Ventres and Wilson, is about “*systematically attending to the historical, social, cultural, political, and economic contexts of host communities. It is the first step toward generating an honest understanding of the root causes of inequity and poor health”*. [47(p34)] To foster such understanding, Reynolds et al [40] suggest that HIC students answer the following reflective questions: *“How will I reconcile the tension between working for health equity while using resources for personal development?”* and *“How can I promote reciprocity between my hosts and other partners?”* and *“Have I discussed ways I could provide benefit to my partners?”* [40(p1108)].

#### Why

Hospital-level reflection aims to build trust and strong relationships with local staff and students while reducing the burden of HIC students’ presence. As Peluso et al. explain, its goal is *“both to benefit students and to provide ongoing feedback to global health faculty as to the adequacy of existing training*” [56(p59)]. Reflection on global inequality, while less tied to the host staff, focuses on HIC students’ transformative learning. Nine articles made mention of ‘transformative learning’, for which the LMIC clinical elective serves as a tool. This was explained in terms of *“fostering the development of global mindedness and community involvement”* [45(p6)], *“working cooperatively in the pursuit of social justice”* [47(p34)], and to *“develop a strong sense of global citizenship”* [48(p3)], and lastly, *“[…] to create a worldview that considers issues such as colonialism, imperialism and systemic social inequality. […] It is essential to understand how the developing world is subjugated by the developed world, historically and today, and how poverty can be reinforced through one’s day-to-day actions”* [19(p8)].

#### Who

HIC medical schools play a central role in facilitating transformative learning and teaching students not only what to reflect upon but also how to reflect. Pre-Departure Training (PDT) and Post-Return Training (PRT) were frequently mentioned, with specific mention of PRT in the context of facilitating and encouraging student reflection and, thus, transformative learning.

#### Implementation and tensions

Although reflective practices are widely promoted, their implementation is largely confined to structured Pre-Departure and Post-Return Training, with limited attention to how reflection is sustained during electives or translated into practice. This narrow framing positions reflection primarily as a tool for individual learning during LMIC electives, while overlooking its potential role as a sustained critical practice for recognizing and addressing health inequities within students’ own HIC contexts as well.

### Theme 3: Local embeddedness

#### What

Local embeddedness, emphasized in nearly all articles, refers to how well HIC students’ work and attitudes align with local expectations during LMIC clinical electives. Three overlapping elements are identified. The first element is ‘appropriate hospital engagement and clinical care’ and encompasses working with and under supervision of local health workers to ensure alignment with clinical practices. The second element is ‘clarity on HIC students’ learning goals and training level’ which is about clear communication to align learning opportunities with local possibilities [41]. The third element is ‘navigation of unequal power dynamics’, to prevent power dynamics hindering local embeddedness of LMIC clinical electives [43,46,58,59].

#### Why

Some health workers in LMIC host hospitals have expressed frustration with visiting students’ clinical care that was impractical or inappropriate to the local context [43]. Ensuring appropriate hospital engagement and context-specific care is vital to avoid such issues and promote safe, effective clinical practice. The clarity on HIC students’ learning goals and training levels is crucial to mitigate risks such as POST and to facilitate good clinical care [40,41,43,44,45,46,47,48,49,51,53,54,56,58,59,60]. Navigating unequal power dynamics is essential to prevent situations where host staff feel disrespected or discriminated against, such as patients overestimating visiting students’ qualifications or preferring their care over that of local trainees [43,58]. Instances of cultural insensitivity by visiting students—like rejecting customary hospitality, inappropriate donations, or neglecting basic greetings—highlight the critical need for culturally sensitive practices [43].

#### Who

HIC medical schools are identified as key actors in implementing strategies for ‘appropriate hospital engagement and clinical care.’ A commonly highlighted approach is Pre-Departure Training to prepare students for differing disease burdens, care standards, and sociocultural norms [40,41,42,43,45,46,47,48,50,52,53]. Suggested organizational strategies include sending fewer students at a time to reduce the burden on local staff [60,61], facilitating longer elective durations [43,46,51,53,62,63], and limiting participation to students with advanced training [43,51,53,60]. Students are also encouraged to engage in cultural immersion and local orientation to better understand the context of staff, students, and patients [45,53,59]. The literature rarely addresses the role of LMIC host hospitals. Only three articles mention involving host hospitals in HIC student screening and selection, emphasizing their insight into a student’s suitability for the local context [43,53,59]. Transparency in selection processes is also recommended [43]. With regard to ‘clarity on HIC students’ learning goals and training level’, HIC students are seen as responsible for communicating their goals. However, further research is needed to align learning goals and opportunities for LMIC clinical electives [41]. For navigating unequal power dynamics, no clear recommendations or responsibilities were identified, possibly reflecting the underrepresentation of host perspectives in the literature.

#### Implementation and tensions

While several HIC organizational strategies are proposed – such as limiting student numbers, extending duration, and improving preparation – there is limited evidence on their implementation or effectiveness. More importantly, there is little attention to how host institutions shape, direct, or meaningfully govern elective activities, suggesting a risk of instrumentalizing ‘the local’ without fully recognizing the agency of host institutions’ staff.

### Theme 4: Equitable partnerships

#### What

Most articles (all but one) highlight the importance of institutional partnerships between sending and hosting institutions, emphasizing sustainability and reciprocity. Sustainability refers to long-term institutional collaboration for structured elective programs. Reciprocity involves three aspects. One is mutual benefit, including recognition and financial compensation for LMIC supervisors [43,53,59]. Second, reciprocity may take the form of ‘involvement of LMIC hosts in program development’, for instance through calling on HIC medical schools to extend the duration of the electives and/or only send advanced-level medical students. Third, reciprocity is about creating exchange opportunities for LMIC students and health workers like those for HIC students.

#### Why

Sustainability enables HIC institutions to better understand and mitigate the risks and burdens of LMIC clinical electives, such as reducing instances of POST [49,52,53,58]. Sustainable partnerships foster accountability, as inappropriate student behavior can be more easily communicated by host hospital representatives to their home institutions’ faculty [48] and they strengthen meaningful relationships at both institutional and individual levels.

One article highlights the benefit of a sustainable partnership to mitigate climate impact [62]. The authors emphasize the value of international clinical electives for medical education, but also call for recognizing the ethical conflict of duty posed by air travel by HIC students, combined with the inequitable burdens of climate damage on LMICs. The authors propose long-term institutional HIC-LMIC partnerships to reorganize electives programs based on the principles of decolonization and decarbonization. This approach includes setting minimum elective durations, supporting host hospitals, and engaging in climate advocacy: *“STEGH agreements could incorporate climate and health-associated infrastructure development as identified by the host institution as a component of the partnership. For example, funding a transition to renewable energy such as solar panels might provide reliable electricity and uninterrupted patient care while also improving air quality, mitigating climate change, and providing health co-benefits. Academic institutions can also deepen their interdepartmental collaboration to support novel host country initiatives. […] In addition, through bidirectional learning, academic medical centers could develop and implement sustainability practices that fit the local context of host countries”* [62(p508)].

Reciprocity in recognition and compensation for LMIC health workers remains critical. Financial or resource contributions by HIC institutions often do not directly benefit supervisors, reducing their incentive to engage with students. This lack of direct compensation can lead to conflicts between responsibilities to local and visiting students [43]. Recognizing individual contributions through direct incentives is essential.

Involving LMIC hosts in program development ensures that their needs and priorities are addressed, resulting in benefits like increased training opportunities, expanded knowledge, satisfaction from teaching, and opportunities for scholarship. Bidirectional exchanges further support career development, networking, and equitable institutional partnerships [40].

#### Who

Based on the articles reviewed, the predominant focus is on the role of HIC institutions. Remarkably, the study results show that HIC institutions can have contrasting ways of dealing with certain risks associated with LMIC electives. For instance, to mitigate the risk of overburdening LMIC staff, in two articles the suggestion was made for HIC staff to accompany their students on their LMIC trips for supervision. In contrast, HIC authors from another article advised against it as their LMIC respondents were not in favour of this practice, and benefits have not been evaluated well [59].

Formalizing institutional relationships can be done by establishing a Memorandum of Understanding to foster meaningful conversations on commitments and priorities. As mentioned by Reynolds et al.: *“Be aware, even formal MOUs require supported, intentional plans for collaboration and accountability, or else risk becoming relics of good intentions. With today’s widespread technological connection, there are numerous ways to structure sustainable partnerships for long-term interconnectedness”* [40(p1109)].

Also clearly addressed is the role for HIC students to transcend the personal level of their own elective experiences, and become actors within the partnership, representatives of the HIC institution, and actively advocate for and contribute to the sustainability of the partnership. One way to do that is by providing feedback that can support the building of equitable partnerships [40,43,48,50,59,61]. Lastly, the role of host hospital staff in building sustainable partnerships is mentioned, e.g. through HIC student supervision, selection and guideline creation [43,44,50,51].

#### Implementation and tensions

While equitable partnerships are widely emphasized, their operationalization often centers on reciprocity, capacity building, and relational continuity, with limited attention to broader structural and planetary dimensions of equity. Only one article explicitly addresses the climate implications of LMIC electives, highlighting the ethical tension between the educational value of international placements and the carbon-intensive nature of air travel by HIC students, alongside the disproportionate burden of climate change on LMICs. The authors propose reconfiguring electives through long-term HIC–LMIC partnerships grounded in principles of decarbonisation and decolonisation, including minimum elective durations, institutional investment in host settings, and engagement in climate advocacy. However, such considerations remain largely absent from the broader literature, suggesting that current conceptions of “equitable partnerships” insufficiently account for climate justice and its implications for global health education.

### Theme 5: Student health and well-being

#### What

Students’ mental and physical well-being is critical in LMIC clinical electives, with risks including emotional distress, guilt over limited care, and culture shock in low-resource settings [41,43,45,48,49,50,52,53,57,58,60,62]. For some students, such challenges can lead to traumatic experiences [49] and poor long-term coping [58].

#### Why

Prioritizing students’ health and well-being is essential to ensure their safety during activities, protect patient care, and avoid straining local health workforce capacity. A significant risk to both students and patients is practicing outside scope of training (POST), which is more common in understaffed rural clinics than in larger academic centers in LMICs. Causes of POST include a mismatch of HIC students’ skills and host expectations, suboptimal supervision at host sites, poor preparation to refuse POST, perceived lack of alternatives, and emergencies. POST often results in lasting moral distress for students [49]. When local health workers must manage visiting students’ well-being amid staff shortages, it strains resources and may impact patient care. Conflicts over care standards, especially in settings of extreme poverty or patient suffering, further burden local staff and compromise workforce capacity.

#### Who

According to the articles included in the review, the responsibility for student health and well-being primarily lies with HIC institutions. There are two crucial moments: *pre-departure*, to prepare students for managing expectations and culture shock in low-resource settings, and *post-return*, to help process difficult experiences and emotions. Addressing POST involves both preparation and debriefing, particularly for those exposed to traumatic events, emphasizing the need for emotional and moral support [49(p8)]. Post-return training also aids educators in identifying students needing further resources and fosters reflection and transformative learning [58].

Shared responsibility between sending and hosting institutions is noted for emergency preparedness, ensuring students have access to mental health resources and familiarity with them before departure. Regular check-ins during rotations are considered critical [57]. Lastly, host institutions also play a role in student well-being through adequate supervision and providing guidelines, though it is unclear whether these should focus on clinical, behavioral, or cultural aspects.

#### Implementation and tensions

While student well-being is consistently emphasized, responsibility is predominantly placed on HIC institutions, with limited consideration of the burden this may place on host institutions or how support is coordinated in practice. In addition, the focus is predominantly on HIC students, with comparatively limited attention to the well-being of LMIC supervisors and healthcare workers, highlighting an imbalance in whose vulnerabilities are recognized and prioritized.

## Discussion

This systematic review examines ethical guidance for medical schools and students in HICs conducting clinical electives in LMICs by analyzing both key themes (what is emphasized), distinguishing between ethical student conduct and the ethics of elective programs, and examining knowledge production through pose and gaze. Our findings contribute to the ongoing dialogue on social justice in medical education by highlighting epistemic and structural imbalances in the development and implementation of ethical guidance, as well as the limited translation of these principles into practice.

Recent literature remains predominantly authored by HIC researchers, primarily from North American institutions (pose), for HIC audiences (gaze). While English remains the dominant language of academic publication, which may present challenges for some LMIC authors, linguistic and structural factors, such as access to funding, mentorship, and publication networks, are all important in shaping authorship patterns. These factors collectively reflect and reinforce epistemic injustice, shaping whose knowledge is produced, recognized, and acted upon [39,64,65,66]. While some LMIC actors are acknowledged—primarily in roles related to student supervision, selection, and guideline creation—it remains unclear whether these align with LMIC actors’ priorities. Our findings suggest that ethical guidance often assigns responsibilities to LMIC institutions rather than being shaped by them. Further research is needed to determine how LMIC institutions can play a more central role in defining and operationalizing ethical guidance. Specifically, future research should encourage scholarly engagement from LMICs themselves to co-create guidance, fostering meaningful partnerships and increasing the manuscript’s impact beyond HIC settings. Moreover, our study is part of a broader transdisciplinary research initiative conducted with partners in Malawi, Suriname, and Ghana (the manuscript ‘A collaborative and reciprocal framework to build equitable partnerships for international medical electives’ is pending at Discover Public Health), aimed at developing contextually relevant and mutually beneficial frameworks for international clinical electives. These collaborations strengthen the call for LMIC-led research and highlight the importance of equitable, co-created ethical guidance.

We identified five core themes in ethical guidance: 1) humility, 2) reflection, 3) local embeddedness, 4) equitable partnerships, and 5) student health and well-being. Themes related to ethical student conduct reinforce established frameworks, such as those of Pinto & Upshur. For example, the principle of humility aligns with their argument that being in an unfamiliar setting creates a disadvantage, forming a foundation for learning. Similarly, our theme of reflection echoes their principles of introspection and social justice, despite differences in terminology.

However, ethical guidance does not only concern student conduct. Our findings distinguish between ethical HIC student conduct and ethical international elective programs. Across themes, ethical responsibility is located predominantly at the level of individual students, while institutional responsibilities – particularly those of HIC institutions – remain less clearly articulated. Historically, ethical guidance literature has focused on ensuring students act ethically during electives—an approach that assigns responsibilities to HIC students, medical schools, and LMIC receiving institutions. Pinto & Upshur exemplify this approach, stating that “teachers and institutions have a responsibility to provide training in ethics,” for which they developed a student-oriented framework [19(p2)]. This focus assigns roles to HIC students, HIC medical schools, and LMIC receiving institutions, ensuring students act ethically during electives. However, in the past five years, a shift toward institutional ethics has gained traction. This approach recognizes that program design itself – rather than just student behavior – can perpetuate inequities. It questions whether HIC medical school programs are ethically justifiable, even if students act ethically within them. Expanding the focus from individual conduct to institutional responsibility is essential for shaping the future of international clinical electives.

While four of the five themes relate to ethical student conduct, their practical implementation requires institutional commitment. Local embeddedness, for example, ensures student learning goals align with hospital expectations, but this alignment depends on both HIC medical schools (e.g., through placement management and Pre-Departure Training) and LMIC institutions (e.g., through structured supervision and integration). Despite widespread agreement in the literature on guiding principles, what remains lacking are actionable guidelines that ensure accountability – for both students and institutions. Ethical frameworks often outline broad principles without specifying how these should be enforced or who is responsible for ensuring adherence, nor how such principles are sustained in practice beyond Pre-Departure and Post-Return Training.

The principle of equitable partnerships differs from the other themes because it addresses the meso-level of institutional ethics rather than just individual student conduct. It emphasizes reciprocity and sustainability in line with broader global health debates [3,33,34,35,36,67,68,69]. While the literature increasingly acknowledges this principle, guidance on operationalizing equitable partnerships remains vague. It is unclear to what extent HIC institutions are committed to transforming traditional, unidirectional electives into bidirectional partnerships grounded in social justice. Moreover, current conceptualizations of equitable partnerships rarely engage with broader structural dimensions, such as climate justice, respite their relevance to global health. The absence of clear accountability mechanisms suggests that this remains largely aspirational rather than systematically implemented. Strengthening evaluation frameworks could help assess partnership effectiveness, enhance mutual accountability [36] and identify actionable strategies. We echo Cummings et al.’s call to give epistemic attention to those involved but unheard [64].

Sustainability is another underdeveloped area in the literature. While some studies frame sustainability as a process, they rarely address ecological sustainability. Despite the urgency of the climate crisis and its disproportionate impact on LMICs, these dimensions remain absent from ethical guidance on international clinical electives. From a social and climate justice perspective, academic institutions must confront these dilemmas and reconsider whether, and under what conditions, international electives should continue to be promoted [70,71,72].

### Strengths and limitations

This systematic review of ethical guidance for HIC medical students in LMIC clinical electives has two main contributions. First, by examining both thematic outcomes (what is considered important) and knowledge production processes (who produces it), the study addresses authorship imbalances as epistemic injustice, linking it to social justice. Second, beyond identifying key ethical themes, this review highlights the lack of concrete, actionable principles that translate ethical guidance into practice. While ethical guidelines emphasize broad virtues and responsibilities, our findings underscore a gap in operational guidance, particularly regarding institutional accountability and program design. By making this distinction, our study contributes to ongoing discussions on how to move from ethical principles to ethical practice.

Our study has several limitations. First, we focused on medical students in HICs, excluding residents, junior doctors, qualified health workers, and volunteers abroad, to avoid confusion with data not specific to medical students’ clinical electives. While the identified themes may apply to other health students and healthcare workers, we cannot confirm this. Second, we included only English-language articles, which may have excluded relevant publications from LMIC authors and reinforces the linguistic bias discussed in this review. A related limitation is that literature from non-Western high-income settings (e.g., Japan, Singapore, and South Korea) may also be underrepresented, reflecting the broader dominance of Western perspectives in global health education discourse. Fourth, the review was restricted to selected databases and did not systematically include grey literature or institutional reports from LMIC institutions, which could have provided additional perspectives and insights given the low publication rate from the global south. A fifth limitation is that the search strategy was intentionally broad and developed with support from a university librarian; however, this involved trade-offs between sensitivity and specificity, potentially leading to both inclusion of less relevant records and omission of relevant HIC contexts not captured by country-level terms. This challenge reflects an inherent limitation in database-driven search strategies for heterogeneous and globally distributed ethical literature. The search was conducted in October 2023; consequently, literature published after this date was not included. A seventh limitation relates to the categorization of authorship based on institutional affiliation. While we categorized authors as HIC- or LMIC-affiliated, this binary distinction does not capture the complexity of transnational academic trajectories, such as LMIC scholars working in HIC institutions or vice versa. A more nuanced approach to authorship categorization—considering career mobility and institutional power structures—could provide a deeper understanding of knowledge production dynamics [73]. Finally, while sustainability is briefly acknowledged in some ethical guidance, its operationalization remains absent. Our study did not systematically analyze the role of environmental sustainability in clinical electives, yet this is an emerging concern in global health. Future research should explore how ecological responsibility can be integrated into ethical frameworks for international medical training.

## Conclusion

This systematic review identifies five interconnected themes in ethical guidance for medical students and their medical schools in HICs for clinical electives in LMICs: 1) humility, 2) reflection, 3) local embeddedness, 4) equitable partnerships, and 5) student health and well-being. We distinguish between ‘ethical HIC student conduct’ and ‘ethical international elective programs,’ noting that most themes emphasize student conduct. However, our findings highlight that ethical guidance remains largely conceptual, with limited focus on how ethical principles can be operationalized. Only equitable partnerships challenge program structures, focusing on reciprocity and sustainability principles.

To advance international clinical electives, greater attention is needed for institutional accountability and the development of actionable principles that move beyond broad ethical ideals. Current guidance predominantly locates responsibility at the level of HIC students, while underemphasizing the role of HIC institutions and the agency of LMIC partners in shaping, governing, and evaluating elective programs. Ethical responsibility should not be associated solely with HIC student conduct, but also with HIC and LMIC institutions’ structuring programs to foster equity, sustainability, and mutual benefit. Future efforts should prioritize co-developing ethical frameworks that are contextually relevant, collaboratively owned, and practically implementable, with active engagement of LMIC partners to ensure mutually beneficial and contextually relevant guidance. In addition, the intersection of ethics and sustainability should be addressed explicitly, considering both social and ecological justice.

## Additional File

The additional file for this article can be found as follows:

10.5334/pme.2150.s1 Supplementary Material. Search Strings.
