# Supplementary material for: Ethical Guidance for Medical Schools and Medical Students in High-Income Countries for Clinical Electives in Low- and Middle-Income Countries: A Systematic Review
Source: Perspect Med Educ. 2026 Jul 14;15(1):600–20. doi: 10.5334/pme.2150 (PMC13378422; doi:10.5334/pme.2150)
Supplement: Supplementary Material. — Search Strings. [file pme-15-1-2150-s1.pdf]

Supplementary Material: Search Strings

PubMed

(((("receiving communit*"[Title/Abstract] OR "local communit*"[Title/Abstract] OR "host communit*"[Title/Abstract] OR "local population*"[Title/Abstract] OR "abroad*"[Title/Abstract] OR "low resource setting*"[Title/Abstract] OR "resource limited setting*"[Title/Abstract] OR "low- and middle income countr*"[Title/Abstract] OR "developing econom*"[Title/Abstract] OR "less developed econom*"[Title/Abstract] OR "under developed econom*"[Title/Abstract] OR "underdeveloped econom*"[Title/Abstract] OR "middle income econom*"[Title/Abstract] OR "low income econom*"[Title/Abstract] OR "underserved econom*"[Title/Abstract] OR "under served econom*"[Title/Abstract] OR "deprived econom*"[Title/Abstract] OR "poor econom*"[Title/Abstract] OR "developing world"[Title/Abstract] OR "less developed world"[Title/Abstract] OR "under developed world"[Title/Abstract] OR "middle income world"[Title/Abstract] OR "low income world"[Title/Abstract] OR "underserved world"[Title/Abstract] OR "under served world"[Title/Abstract] OR "deprived world"[Title/Abstract] OR "poor world"[Title/Abstract] OR "developing countr*"[Title/Abstract] OR "less developed countr*"[Title/Abstract] OR "under developed countr*"[Title/Abstract] OR "underdeveloped countr*"[Title/Abstract] OR "middle income countr*"[Title/Abstract] OR "low income countr*"[Title/Abstract] OR "underserved countr*"[Title/Abstract] OR "under served countr*"[Title/Abstract] OR "deprived countr*"[Title/Abstract] OR "poor countr*"[Title/Abstract] OR "developing nation"[Title/Abstract] OR "less developed nation"[Title/Abstract] OR "under developed nation"[Title/Abstract] OR "underdeveloped nation"[Title/Abstract] OR "middle income nation"[Title/Abstract] OR "low income nation"[Title/Abstract] OR "underserved nation"[Title/Abstract] OR "under served nation"[Title/Abstract] OR "deprived nation"[Title/Abstract] OR "poor nation"[Title/Abstract] OR "developing population"[Title/Abstract] OR "less developed population"[Title/Abstract] OR "under developed population"[Title/Abstract] OR "underdeveloped population"[Title/Abstract] OR "middle income population"[Title/Abstract] OR "low income population"[Title/Abstract] OR "underserved population"[Title/Abstract] OR "under served population"[Title/Abstract] OR "deprived population"[Title/Abstract] OR "poor population"[Title/Abstract] OR "low gdp"[Title/Abstract] OR "low gnp"[Title/Abstract] OR "low gross domestic"[Title/Abstract] OR "low gross national"[Title/Abstract] OR "low middle income countr*"[Title/Abstract] OR "lmic*"[Title/Abstract] OR "third world"[Title/Abstract] OR "lami countr*"[Title/Abstract] OR "transitional countr*"[Title/Abstract] OR "developing countr*"[Title/Abstract] OR "global south"[Title/Abstract] OR "global north and south"[Title/Abstract] OR "africa south of the sahara"[Title/Abstract] OR "africa, central"[Title/Abstract] OR "central africa"[Title/Abstract] OR "africa, eastern"[Title/Abstract] OR "eastern africa"[Title/Abstract] OR "africa, southern"[Title/Abstract] OR "southern africa"[Title/Abstract] OR "africa, western"[Title/Abstract] OR "western africa"[Title/Abstract] OR "sub-saharan africa"[Title/Abstract] OR "Afghanistan"[Title/Abstract] OR "Albania"[Title/Abstract] OR "Algeria"[Title/Abstract] OR "Angola"[Title/Abstract] OR "Argentina"[Title/Abstract] OR "Armenia"[Title/Abstract] OR "Azerbaijan"[Title/Abstract] OR "Bangladesh"[Title/Abstract] OR "Republic of Belarus"[Title/Abstract] OR "belarus"[Title/Abstract] OR "byelarus"[Title/Abstract] OR "belorussia"[Title/Abstract] OR "Belize"[Title/Abstract] OR "Benin"[Title/Abstract] OR "Dahomey"[Title/Abstract] OR "Bhutan"[Title/Abstract] OR "Bolivia"[Title/Abstract] OR "Bosnia-Herzegovina"[Title/Abstract] OR "Bosnia"[Title/Abstract] OR "Botswana"[Title/Abstract] OR "Bechuanaland"[Title/Abstract]

OR "Kalahari"[Title/Abstract] OR "Brazil"[Title/Abstract] OR "Bulgaria"[Title/Abstract] OR "Burkina Faso"[Title/Abstract] OR "Burkina Fasso"[Title/Abstract] OR "Upper Volta"[Title/Abstract] OR "Burundi"[Title/Abstract] OR "Cape Verde"[Title/Abstract] OR "Cabo Verde"[Title/Abstract] OR "Cambodia"[Title/Abstract] OR "Cameroon"[Title/Abstract] OR "Central African Republic"[Title/Abstract] OR "Central African Republic"[Title/Abstract] OR "Ubangi-Shari"[Title/Abstract] OR "Chad"[Title/Abstract] OR "China"[Title/Abstract] OR "Colombia"[Title/Abstract] OR "Comoros"[Title/Abstract] OR "Comoro Islands"[Title/Abstract] OR "Mayotte"[Title/Abstract] OR "Iles Comores"[Title/Abstract] OR ("Congo"[Title/Abstract] NOT (congo red[Title/Abstract] OR crimean-congo[Title/Abstract])) OR "Costa Rica"[Title/Abstract] OR "Ivory Coast"[Title/Abstract] OR "Cote d'Ivoire"[Title/Abstract] OR "Ivory Coast"[Title/Abstract] OR "Cuba"[Title/Abstract] OR "Democratic Republic of the Congo"[Title/Abstract] OR "Belgian Congo"[Title/Abstract] OR "Zaire"[Title/Abstract] OR "Democratic People's Republic of Korea"[Title/Abstract] OR "North Korea"[Title/Abstract] OR "Democratic People Republic Korea"[Title/Abstract] OR "Djibouti"[Title/Abstract] OR "French Somaliland"[Title/Abstract] OR "Dominica"[Title/Abstract] OR "Dominican Republic"[Title/Abstract] OR "Ecuador"[Title/Abstract] OR "Egypt"[Title/Abstract] OR "El Salvador"[Title/Abstract] OR "Equatorial Guinea"[Title/Abstract] OR "Eritrea"[Title/Abstract] OR "Ethiopia"[Title/Abstract] OR "Eswatini"[Title/Abstract] OR "Fiji"[Title/Abstract] OR "Gabon"[Title/Abstract] OR "Gabonese Republic"[Title/Abstract] OR "Gambia"[Title/Abstract] OR "Georgia"[Title/Abstract] OR "Ghana"[Title/Abstract] OR "Gold Coast"[Title/Abstract] OR "Guatemala"[Title/Abstract] OR ("Guinea"[Title/Abstract] NOT ("New Guinea"[Title/Abstract] OR "Guinea Pig*"[Title/Abstract] OR "Guinea Fowl"[Title/Abstract])) OR "Guinea-Bissau"[Title/Abstract] OR "Portuguese Guinea"[Title/Abstract] OR "Haiti"[Title/Abstract] OR "Honduras"[Title/Abstract] OR "India"[Title/Abstract] OR "Indonesia"[Title/Abstract] OR "Iran"[Title/Abstract] OR "Iraq"[Title/Abstract] OR "Jamaica"[Title/Abstract] OR "Jordan"[Title/Abstract] OR "Kazakhstan"[Title/Abstract] OR "Kazakh"[Title/Abstract] OR "Kenya"[Title/Abstract] OR "Kiribati"[Title/Abstract] OR "Kyrgyzstan"[Title/Abstract] OR "Kyrgyz Republic"[Title/Abstract] OR "Kirghizia"[Title/Abstract] OR "Kirghiz"[Title/Abstract] OR "Laos"[Title/Abstract] OR "Lao Democratic Republic"[Title/Abstract] OR "Lebanon"[Title/Abstract] OR "Lesotho"[Title/Abstract] OR "Basutoland"[Title/Abstract] OR "Liberia"[Title/Abstract] OR "Libya"[Title/Abstract] OR "Madagascar"[Title/Abstract] OR "Malagasy Republic"[Title/Abstract] OR "Malawi"[Title/Abstract] OR "Nyasaland"[Title/Abstract] OR "Malaysia"[Title/Abstract] OR "Maldives"[Title/Abstract] OR "Mali"[Title/Abstract] OR "Marshall Islands"[Title/Abstract] OR "Mauritania"[Title/Abstract] OR "Mauritius"[Title/Abstract] OR "Agalega Islands"[Title/Abstract] OR "Mexico"[Title/Abstract] OR "Micronesia"[Title/Abstract] OR "Moldova"[Title/Abstract] OR "Mongolia"[Title/Abstract] OR "Montenegro"[Title/Abstract] OR "Morocco"[Title/Abstract] OR "Mozambique"[Title/Abstract] OR "Portuguese East Africa"[Title/Abstract] OR "Myanmar"[Title/Abstract] OR "Burma"[Title/Abstract] OR "Namibia"[Title/Abstract] OR "Nepal"[Title/Abstract] OR "Nicaragua"[Title/Abstract] OR ("Niger"[Title/Abstract] NOT ("Aspergillus"[Title/Abstract] OR "Peptococcus"[Title/Abstract] OR "Schizothorax"[Title/Abstract] OR "Cruciferae"[Title/Abstract] OR "Gobius"[Title/Abstract] OR "Lasius"[Title/Abstract] OR "Agelastes"[Title/Abstract] OR "Melanosuchus"[Title/Abstract] OR "radish"[Title/Abstract] OR "Parastromateus"[Title/Abstract] OR "Orius"[Title/Abstract] OR "Parastromateus"[Title/Abstract] OR "Stomoxys"[Title/Abstract])) OR "Nigeria"[Title/Abstract] OR "Macedonia (Republic)"[Title/Abstract] OR "Macedonia"[Title/Abstract] OR "Pakistan"[Title/Abstract] OR "Palau"[Title/Abstract] OR

"Papua New Guinea"[Title/Abstract] OR "Paraguay"[Title/Abstract] OR "Peru"[Title/Abstract] OR "Philippines"[Title/Abstract] OR "Russia"[Title/Abstract] OR "Russian Federation"[Title/Abstract] OR "Rwanda"[Title/Abstract] OR "Ruanda"[Title/Abstract] OR "Samoa"[Title/Abstract] OR "independent state of samoa"[Title/Abstract] OR "Senegal"[Title/Abstract] OR "Serbia"[Title/Abstract] OR "Sierra Leone"[Title/Abstract] OR "Solomon Islands"[Title/Abstract] OR "Somalia"[Title/Abstract] OR "South Africa"[Title/Abstract] OR "Sri Lanka"[Title/Abstract] OR "Saint Lucia"[Title/Abstract] OR "Saint Vincent the Grenadines"[Title/Abstract] OR "Palestine"[Title/Abstract] OR "South Sudan"[Title/Abstract] OR "Sudan"[Title/Abstract] OR "Suriname"[Title/Abstract] OR "Syria"[Title/Abstract] OR "Syrian Arab Republic"[Title/Abstract] OR "Tajikistan"[Title/Abstract] OR "Tadzhik"[Title/Abstract] OR "Tadzhikistan"[Title/Abstract] OR "Tajikistan"[Title/Abstract] OR "Thailand"[Title/Abstract] OR "Timor-Leste"[Title/Abstract] OR "Togo"[Title/Abstract] OR "Togolese Republic"[Title/Abstract] OR "Tonga"[Title/Abstract] OR "Tunisia"[Title/Abstract] OR ("Turkey"[Title/Abstract] NOT animal[Title/Abstract]) OR "Turkiye"[Title/Abstract] OR "Turkmenistan"[Title/Abstract] OR "Tuvalu"[Title/Abstract] OR "Uganda"[Title/Abstract] OR "Ukraine"[Title/Abstract] OR "Tanzania"[Title/Abstract] OR "Zanzibar"[Title/Abstract] OR "Uzbekistan"[Title/Abstract] OR "Vanuatu"[Title/Abstract] OR "Vietnam"[Title/Abstract] OR "West Bank"[Title/Abstract] OR "Gaza"[Title/Abstract] OR "Yemen"[Title/Abstract] OR "Zambia"[Title/Abstract] OR "Northern Rhodesia"[Title/Abstract] OR "Zimbabwe"[Title/Abstract] OR "Rhodesia"[Title/Abstract] OR "Yugoslavia"[Title/Abstract] OR "Kosovo"[Title/Abstract] OR "Indian Ocean Islands"[Title/Abstract]) OR (Developing Countries[MeSH Terms])) AND (((((("medical student*"[Title/Abstract] OR "med-student*"[Title/Abstract] OR "medical graduate student*"[Title/Abstract] OR "graduate medical student*"[Title/Abstract] OR "medical undergraduate student*"[Title/Abstract] OR "undergraduate medical student*"[Title/Abstract] OR "Western med student*"[Title/Abstract] OR "Western medical student*"[Title/Abstract] OR "student*"[Title/Abstract] OR "trainee*"[Title/Abstract] OR "medical trainee*"[Title/Abstract] OR "clinical trainee*"[Title/Abstract] OR "learner*"[Title/Abstract] OR "intern*"[Title/Abstract] OR "candidate*"[Title/Abstract] OR "apprentice*"[Title/Abstract] OR "undergraduate medical education"[Title/Abstract] OR "graduate medical education"[Title/Abstract] OR "Western student"[Title/Abstract] OR "internship and residenc*" OR[Title/Abstract]) OR ("academic medical center*"[Title/Abstract] OR "academic healthcare*"[Title/Abstract] OR "medical institution*"[Title/Abstract] OR "medical school*"[Title/Abstract] OR "med-school*"[Title/Abstract] OR "medical facult*"[Title/Abstract] OR "medical curricul*"[Title/Abstract] OR "medical education"[Title/Abstract] OR "curriculum evaluation*"[Title/Abstract] OR "academic curriculum evaluation*"[Title/Abstract] OR "sending institution*"[Title/Abstract] OR "host hospital*"[Title/Abstract] OR "host institution*"[Title/Abstract] OR "sending hospital*"[Title/Abstract])) OR (students, medical[MeSH Terms])) OR (Education, Medical, Graduate[MeSH Terms])) OR (education, medical, undergraduate[MeSH Terms])) OR (academic medical centers[MeSH Terms]))) AND ((("ethic*"[Title/Abstract] OR "moral*"[Title/Abstract] OR "value*"[Title/Abstract] OR "responsib*"[Title/Abstract] OR "ethical guid*"[Title/Abstract] OR "guidance"[Title/Abstract] OR "guideline*"[Title/Abstract] OR "conscience"[Title/Abstract] OR "requirement*"[Title/Abstract] OR "duty"[Title/Abstract] OR "sense of duty"[Title/Abstract] OR "duties"[Title/Abstract] OR "obligation*"[Title/Abstract] OR "medical ethic*"[Title/Abstract] OR "professional ethic*"[Title/Abstract] OR "post-colonial ethic*"[Title/Abstract] OR "postcolonial ethic*"[Title/Abstract] OR "neo-colonial ethic*"[Title/Abstract] OR "neocolonial ethic*"[Title/Abstract] OR "biomedical

ethic*"[Title/Abstract] OR "bioethic*"[Title/Abstract] OR "bio-ethic*"[Title/Abstract] OR "responsib*"[Title/Abstract] OR "liab*"[Title/Abstract] OR "accountable"[Title/Abstract] OR "answerab*"[Title/Abstract] OR "principl*"[Title/Abstract] OR "justified"[Title/Abstract] OR "justification"[Title/Abstract] OR "pre-departure training"[Title/Abstract] OR "predeparture training"[Title/Abstract] OR "post-return"[Title/Abstract] OR "postreturn"[Title/Abstract]) OR (bioethics[MeSH Terms])) OR (guidelines as topic[MeSH Terms]))) AND ((("International educational exchang*"[Title/Abstract] OR "global health stud*"[Title/Abstract] OR"global health elective*"[Title/Abstract] OR "global health educat*"[Title/Abstract] OR "global health program*"[Title/Abstract] OR "global health intern*"[Title/Abstract] OR "medical elective*"[Title/Abstract] OR "medical volunteer*"[Title/Abstract] OR "international apprenticeship*"[Title/Abstract] OR "international health elective*"[Title/Abstract] OR "international intern*"[Title/Abstract] OR "international clinical clerkship*"[Title/Abstract] OR "international clinical rotation*"[Title/Abstract] OR "international clinical experience*"[Title/Abstract] OR "international rotation*"[Title/Abstract] OR "practical training"[Title/Abstract] OR "short-term international trip*"[Title/Abstract] OR "clinical clerkship*"[Title/Abstract] OR "clinical elective*"[Title/Abstract] OR "international medical traineeship*"[Title/Abstract] OR "short-term experience*"[Title/Abstract] OR "short-term international medical initiative*"[Title/Abstract] OR "short-term medical initiative*"[Title/Abstract] OR "international medical initiative*"[Title/Abstract] OR "international medical endeavor*"[Title/Abstract] OR "global health educational opportunit*"[Title/Abstract] OR "global health training*"[Title/Abstract] OR "global health practicum*"[Title/Abstract] OR "global health experience*"[Title/Abstract] OR "global health outreach work"[Title/Abstract] OR "service learning"[Title/Abstract] OR "international clinical education*"[Title/Abstract] OR "medical mission*"[Title/Abstract] OR "global health mission*"[Title/Abstract] OR "mission trip*"[Title/Abstract] OR "bidirectional exchange*"[Title/Abstract]) OR ("International educational exchange"[MeSH Terms])) OR ("Internship and Residency"[MeSH Terms]))

Embase

"receiving communit*":ti,ab,kw OR "local communit*":ti,ab,kw OR "host communit*":ti,ab,kw OR "local population*":ti,ab,kw OR "abroad*":ti,ab,kw OR "low resource setting*":ti,ab,kw OR "resource limited setting*":ti,ab,kw OR "low- and middle income countr*":ti,ab,kw OR "developing econom*":ti,ab,kw OR "less developed econom*":ti,ab,kw OR "under developed econom*":ti,ab,kw OR "underdeveloped econom*":ti,ab,kw OR "middle income econom*":ti,ab,kw OR "low income econom*":ti,ab,kw OR "underserved econom*":ti,ab,kw OR "under served econom*":ti,ab,kw OR "deprived econom*":ti,ab,kw OR "poor econom*":ti,ab,kw OR "developing world":ti,ab,kw OR "less developed world":ti,ab,kw OR "under developed world":ti,ab,kw OR "middle income world":ti,ab,kw OR "low income world":ti,ab,kw OR "underserved world":ti,ab,kw OR "under served world":ti,ab,kw OR "deprived world":ti,ab,kw OR "poor world":ti,ab,kw OR "developing countr*":ti,ab,kw OR "less developed countr*":ti,ab,kw OR "under developed countr*":ti,ab,kw OR "underdeveloped countr*":ti,ab,kw OR "middle income countr*":ti,ab,kw OR "low income countr*":ti,ab,kw OR "underserved countr*":ti,ab,kw OR "under served countr*":ti,ab,kw OR "deprived countr*":ti,ab,kw OR "poor countr*":ti,ab,kw OR "developing nation":ti,ab,kw OR "less developed nation":ti,ab,kw OR "under developed nation":ti,ab,kw OR "underdeveloped nation":ti,ab,kw OR "middle income nation":ti,ab,kw OR "low income nation":ti,ab,kw OR "underserved nation":ti,ab,kw OR "under served nation":ti,ab,kw OR "deprived

nation":ti,ab,kw OR "poor nation":ti,ab,kw OR "developing population":ti,ab,kw OR "less developed population":ti,ab,kw OR "under developed population":ti,ab,kw OR "underdeveloped population":ti,ab,kw OR "middle income population":ti,ab,kw OR "low income population":ti,ab,kw OR "underserved population":ti,ab,kw OR "under served population":ti,ab,kw OR "deprived population":ti,ab,kw OR "poor population":ti,ab,kw OR "low gdp":ti,ab,kw OR "low gnp":ti,ab,kw OR "low gross domestic":ti,ab,kw OR "low gross national":ti,ab,kw OR "low middle income countr*":ti,ab,kw OR "lmic*":ti,ab,kw OR "third world":ti,ab,kw OR "lami countr*":ti,ab,kw OR "transitional countr*":ti,ab,kw OR "developing countr*":ti,ab,kw OR "global south":ti,ab,kw OR "global north and south":ti,ab,kw OR "africa south of the sahara":ti,ab,kw OR "africa, central":ti,ab,kw OR "central africa":ti,ab,kw OR "africa, eastern":ti,ab,kw OR "eastern africa":ti,ab,kw OR "africa, southern":ti,ab,kw OR "southern africa":ti,ab,kw OR "africa, western":ti,ab,kw OR "western africa":ti,ab,kw OR "sub-saharan africa":ti,ab,kw OR "Afghanistan":ti,ab,kw OR "Albania":ti,ab,kw OR "Algeria":ti,ab,kw OR "Angola":ti,ab,kw OR "Argentina":ti,ab,kw OR "Armenia":ti,ab,kw OR "Azerbaijan":ti,ab,kw OR "Bangladesh":ti,ab,kw OR "Republic of Belarus":ti,ab,kw OR "belarus":ti,ab,kw OR "byelarus":ti,ab,kw OR "belorussia":ti,ab,kw OR "Belize":ti,ab,kw OR "Benin":ti,ab,kw OR "Dahomey":ti,ab,kw OR "Bhutan":ti,ab,kw OR "Bolivia":ti,ab,kw OR "Bosnia-Herzegovina":ti,ab,kw OR "Bosnia":ti,ab,kw OR "Botswana":ti,ab,kw OR "Bechuanaland":ti,ab,kw OR "Kalahari":ti,ab,kw OR "Brazil":ti,ab,kw OR "Bulgaria":ti,ab,kw OR "Burkina Faso":ti,ab,kw OR "Burkina Fasso":ti,ab,kw OR "Upper Volta":ti,ab,kw OR "Burundi":ti,ab,kw OR "Cape Verde":ti,ab,kw OR "Cabo Verde":ti,ab,kw OR "Cambodia":ti,ab,kw OR "Cameroon":ti,ab,kw OR "Central African Republic":ti,ab,kw OR "Central African Republic":ti,ab,kw OR "Ubangi-Shari":ti,ab,kw OR "Chad":ti,ab,kw OR "China":ti,ab,kw OR "Colombia":ti,ab,kw OR "Comoros":ti,ab,kw OR "Comoro Islands":ti,ab,kw OR "Mayotte":ti,ab,kw OR "Iles Comores":ti,ab,kw OR ("Congo":ti,ab,kw NOT (congo red:ti,ab,kw OR crimean-congo:ti,ab,kw)) OR "Costa Rica":ti,ab,kw OR "Ivory Coast":ti,ab,kw OR "Cuba":ti,ab,kw OR "Democratic Republic of the Congo":ti,ab,kw OR "Belgian Congo":ti,ab,kw OR "Zaire":ti,ab,kw OR "Democratic People Republic of Korea":ti,ab,kw OR "North Korea":ti,ab,kw OR "Democratic People Republic Korea":ti,ab,kw OR "Djibouti":ti,ab,kw OR "French Somaliland":ti,ab,kw OR "Dominica":ti,ab,kw OR "Dominican Republic":ti,ab,kw OR "Ecuador":ti,ab,kw OR "Egypt":ti,ab,kw OR "El Salvador":ti,ab,kw OR "Equatorial Guinea":ti,ab,kw OR "Eritrea":ti,ab,kw OR "Ethiopia":ti,ab,kw OR "Eswatini":ti,ab,kw OR "Fiji":ti,ab,kw OR "Gabon":ti,ab,kw OR "Gabonese Republic":ti,ab,kw OR "Gambia":ti,ab,kw OR "Georgia":ti,ab,kw OR "Ghana":ti,ab,kw OR "Gold Coast":ti,ab,kw OR "Guatemala":ti,ab,kw OR ("Guinea":ti,ab,kw NOT ("New Guinea":ti,ab,kw OR "Guinea Pig*":ti,ab,kw OR "Guinea Fowl":ti,ab,kw)) OR "Guinea-Bissau":ti,ab,kw OR "Portuguese Guinea":ti,ab,kw OR "Haiti":ti,ab,kw OR "Honduras":ti,ab,kw OR "India":ti,ab,kw OR "Indonesia":ti,ab,kw OR "Iran":ti,ab,kw OR "Iraq":ti,ab,kw OR "Jamaica":ti,ab,kw OR "Jordan":ti,ab,kw OR "Kazakhstan":ti,ab,kw OR "Kazakh":ti,ab,kw OR "Kenya":ti,ab,kw OR "Kiribati":ti,ab,kw OR "Kyrgyzstan":ti,ab,kw OR "Kyrgyz Republic":ti,ab,kw OR "Kirghizia":ti,ab,kw OR "Kirghiz":ti,ab,kw OR "Laos":ti,ab,kw OR "Lao Democratic Republic":ti,ab,kw OR "Lebanon":ti,ab,kw OR "Lesotho":ti,ab,kw OR "Basutoland":ti,ab,kw OR "Liberia":ti,ab,kw OR "Libya":ti,ab,kw OR "Madagascar":ti,ab,kw OR "Malagasy Republic":ti,ab,kw OR "Malawi":ti,ab,kw OR "Nyasaland":ti,ab,kw OR "Malaysia":ti,ab,kw OR "Maldives":ti,ab,kw OR "Mali":ti,ab,kw OR "Marshall Islands":ti,ab,kw OR "Mauritania":ti,ab,kw OR "Mauritius":ti,ab,kw OR "Agalega Islands":ti,ab,kw OR "Mexico":ti,ab,kw OR "Micronesia":ti,ab,kw OR "Moldova":ti,ab,kw OR "Mongolia":ti,ab,kw OR "Montenegro":ti,ab,kw OR "Morocco":ti,ab,kw OR "Mozambique":ti,ab,kw OR "Portuguese East Africa":ti,ab,kw OR

"Myanmar":ti,ab,kw OR "Burma":ti,ab,kw OR "Namibia":ti,ab,kw OR "Nepal":ti,ab,kw OR "Nicaragua":ti,ab,kw OR ("Niger":ti,ab,kw NOT ("Aspergillus":ti,ab,kw OR "Peptococcus":ti,ab,kw OR "Schizothorax":ti,ab,kw OR "Cruciferae":ti,ab,kw OR "Gobius":ti,ab,kw OR "Lasius":ti,ab,kw OR "Agelastes":ti,ab,kw OR "Melanosuchus":ti,ab,kw OR "radish":ti,ab,kw OR "Parastromateus":ti,ab,kw OR "Orius":ti,ab,kw OR "Parastromateus":ti,ab,kw OR "Stomoxys":ti,ab,kw)) OR "Nigeria":ti,ab,kw OR "Macedonia (Republic)":ti,ab,kw OR "Macedonia":ti,ab,kw OR "Pakistan":ti,ab,kw OR "Palau":ti,ab,kw OR "Papua New Guinea":ti,ab,kw OR "Paraguay":ti,ab,kw OR "Peru":ti,ab,kw OR "Philippines":ti,ab,kw OR "Russia":ti,ab,kw OR "Russian Federation":ti,ab,kw OR "Rwanda":ti,ab,kw OR "Ruanda":ti,ab,kw OR "Samoa":ti,ab,kw OR "independent state of samoa":ti,ab,kw OR "Senegal":ti,ab,kw OR "Serbia":ti,ab,kw OR "Sierra Leone":ti,ab,kw OR "Solomon Islands":ti,ab,kw OR "Somalia":ti,ab,kw OR "South Africa":ti,ab,kw OR "Sri Lanka":ti,ab,kw OR "Saint Lucia":ti,ab,kw OR "Saint Vincent the Grenadines":ti,ab,kw OR "Palestine":ti,ab,kw OR "South Sudan":ti,ab,kw OR "Sudan":ti,ab,kw OR "Suriname":ti,ab,kw OR "Syria":ti,ab,kw OR "Syrian Arab Republic":ti,ab,kw OR "Tajikistan":ti,ab,kw OR "Tadzhik":ti,ab,kw OR "Tadzhikistan":ti,ab,kw OR "Tajikistan":ti,ab,kw OR "Thailand":ti,ab,kw OR "Timor-Leste":ti,ab,kw OR "Togo":ti,ab,kw OR "Togolese Republic":ti,ab,kw OR "Tonga":ti,ab,kw OR "Tunisia":ti,ab,kw OR ("Turkey":ti,ab,kw NOT animal:ti,ab,kw) OR "Turkiye":ti,ab,kw OR "Turkmenistan":ti,ab,kw OR "Tuvalu":ti,ab,kw OR "Uganda":ti,ab,kw OR "Ukraine":ti,ab,kw OR "Tanzania":ti,ab,kw OR "Zanzibar":ti,ab,kw OR "Uzbekistan":ti,ab,kw OR "Vanuatu":ti,ab,kw OR "Vietnam":ti,ab,kw OR "West Bank":ti,ab,kw OR "Gaza":ti,ab,kw OR "Yemen":ti,ab,kw OR "Zambia":ti,ab,kw OR "Northern Rhodesia":ti,ab,kw OR "Zimbabwe":ti,ab,kw OR "Rhodesia":ti,ab,kw OR "Yugoslavia":ti,ab,kw OR "Kosovo":ti,ab,kw OR "Indian Ocean Islands":ti,ab,kw OR "developing country"/mj AND "medical student*":ti,ab,kw OR "med-student*":ti,ab,kw OR "medical graduate student*":ti,ab,kw OR "graduate medical student*":ti,ab,kw OR "medical undergraduate student*":ti,ab,kw OR "undergraduate medical student*":ti,ab,kw OR "Western med student*":ti,ab,kw OR "Western medical student*":ti,ab,kw OR "student*":ti,ab,kw OR "trainee*":ti,ab,kw OR "medical trainee*":ti,ab,kw OR "clinical trainee*":ti,ab,kw OR "learner*":ti,ab,kw OR "intern*":ti,ab,kw OR "candidate*":ti,ab,kw OR "apprentice*":ti,ab,kw OR "undergraduate medical education":ti,ab,kw OR "graduate medical education":ti,ab,kw OR "Western student":ti,ab,kw OR "internship and residenc*":ti,ab,kw OR "academic medical center*":ti,ab,kw OR "academic healthcare*":ti,ab,kw OR "medical institution*":ti,ab,kw OR "medical school*":ti,ab,kw OR "med-school*":ti,ab,kw OR "medical facult*":ti,ab,kw OR "medical curricul*":ti,ab,kw OR "medical education":ti,ab,kw OR "curriculum evaluation*":ti,ab,kw OR "academic curriculum evaluation*":ti,ab,kw OR "sending institution*":ti,ab,kw OR "host hospital*":ti,ab,kw OR "host institution*":ti,ab,kw OR "sending hospital*":ti,ab,kw OR "medical student"/mj OR "medical education"/mj OR "teaching hospital"/mj AND "ethic*":ti,ab,kw OR "moral*":ti,ab,kw OR "value*":ti,ab,kw OR "responsib*":ti,ab,kw OR "ethical guid*":ti,ab,kw OR "guidance":ti,ab,kw OR "guideline*":ti,ab,kw OR "conscience":ti,ab,kw OR "requirement*":ti,ab,kw OR "duty":ti,ab,kw OR "sense of duty":ti,ab,kw OR "duties":ti,ab,kw OR "obligation*":ti,ab,kw OR "medical ethic*":ti,ab,kw OR "professional ethic*":ti,ab,kw OR "post-colonial ethic*":ti,ab,kw OR "postcolonial ethic*":ti,ab,kw OR "neo-colonial ethic*":ti,ab,kw OR "neocolonial ethic*":ti,ab,kw OR "biomedical ethic*":ti,ab,kw OR "bioethic*":ti,ab,kw OR "bio-ethic*":ti,ab,kw OR "responsib*":ti,ab,kw OR "liab*":ti,ab,kw OR "accountable":ti,ab,kw OR "answerab*":ti,ab,kw OR "principl*":ti,ab,kw OR "justified":ti,ab,kw OR "justification":ti,ab,kw OR "pre-departure training":ti,ab,kw OR "predeparture

training":ti,ab,kw OR "post-return":ti,ab,kw OR "postreturn":ti,ab,kw OR "bioethics"/mj OR "practice guideline"/mj AND "International educational exchang*":ti,ab,kw OR "global health stud*":ti,ab,kw OR "global health elective*":ti,ab,kw OR "global health educat*":ti,ab,kw OR "global health program*":ti,ab,kw OR "global health intern*":ti,ab,kw OR "medical elective*":ti,ab,kw OR "medical volunteer*":ti,ab,kw OR "international apprenticeship*":ti,ab,kw OR "international health elective*":ti,ab,kw OR "international intern*":ti,ab,kw OR "international clinical clerkship*":ti,ab,kw OR "international clinical rotation*":ti,ab,kw OR "international clinical experience*":ti,ab,kw OR "international rotation*":ti,ab,kw OR "practical training":ti,ab,kw OR "short-term international trip*":ti,ab,kw OR "clinical clerkship*":ti,ab,kw OR "clinical elective*":ti,ab,kw OR "international medical traineeship*":ti,ab,kw OR "short-term experience*":ti,ab,kw OR "short-term international medical initiative*":ti,ab,kw OR "short-term medical initiative*":ti,ab,kw OR "international medical initiative*":ti,ab,kw OR "international medical endeavor*":ti,ab,kw OR "global health educational opportunit*":ti,ab,kw OR "global health training*":ti,ab,kw OR "global health practicum*":ti,ab,kw OR "global health experience*":ti,ab,kw OR "global health outreach work":ti,ab,kw OR "service learning":ti,ab,kw OR "international clinical education*":ti,ab,kw OR "medical mission*":ti,ab,kw OR "global health mission*":ti,ab,kw OR "mission trip*":ti,ab,kw OR "bidirectional exchange*":ti,ab,kw OR "international cooperation"/mj OR "residency education"/mj AND [embase]/lim

#14 AND [embase]/lim NOT ([embase]/lim AND [medline]/lim)

Global health

((title:("medical student*" OR "med-student*" OR "medical graduate student*" OR "graduate medical student*" OR "medical undergraduate student*" OR "undergraduate medical student*" OR "Western med student*" OR "Western medical student*" OR "student*" OR "trainee*" OR "medical trainee*" OR "clinical trainee*" OR "learner*" OR "intern*" OR "candidate*" OR "apprentice*" OR "undergraduate medical education" OR "graduate medical education" OR "Western student" OR "internship and residenc*" OR "academic medical center*" OR "academic healthcare*" OR "medical institution*" OR "medical school*" OR "med-school*" OR "medical facult*" OR "medical curricul*" OR "medical education" OR "curriculum evaluation*" OR "academic curriculum evaluation*" OR "sending institution*" OR "host hospital*" OR "host institution*" OR "sending hospital*" OR "medical student" OR "medical education" OR "teaching hospital") OR ab:("medical student*" OR "med-student*" OR "medical graduate student*" OR "graduate medical student*" OR "medical undergraduate student*" OR "undergraduate medical student*" OR "Western med student*" OR "Western medical student*" OR "student*" OR "trainee*" OR "medical trainee*" OR "clinical trainee*" OR "learner*" OR "intern*" OR "candidate*" OR "apprentice*" OR "undergraduate medical education" OR "graduate medical education" OR "Western student" OR "internship and residenc*" OR "academic medical center*" OR "academic healthcare*" OR "medical institution*" OR "medical school*" OR "med-school*" OR "medical facult*" OR "medical curricul*" OR "medical education" OR "curriculum evaluation*" OR "academic curriculum evaluation*" OR "sending institution*" OR "host hospital*" OR "host institution*" OR "sending hospital*" OR "medical student" OR "medical education" OR "teaching hospital"))) AND ((title:("ethic*" OR "moral*" OR "value*" OR "responsib*" OR "ethical guid*" OR "guidance" OR "guideline*" OR "conscience" OR "requirement*" OR "duty" OR "sense of

duty" OR "duties" OR "obligation*" OR "medical ethic*" OR "professional ethic*" OR "post-colonial ethic*" OR "postcolonial ethic*" OR "neo-colonial ethic*" OR "neocolonial ethic*" OR "biomedical ethic*" OR "bioethic*" OR "bio-ethic*" OR "responsib*" OR "liab*" OR "accountable" OR "answerab*" OR "principl*" OR "justified" OR "justification" OR "pre-departure training" OR "predeparture training" OR "post-return" OR "postreturn" OR "practice guideline*") OR ab:("ethic*" OR "moral*" OR "value*" OR "responsib*" OR "ethical guid*" OR "guidance" OR "guideline*" OR "conscience" OR "requirement*" OR "duty" OR "sense of duty" OR "duties" OR "obligation*" OR "medical ethic*" OR "professional ethic*" OR "post-colonial ethic*" OR "postcolonial ethic*" OR "neo-colonial ethic*" OR "neocolonial ethic*" OR "biomedical ethic*" OR "bioethic*" OR "bio-ethic*" OR "responsib*" OR "liab*" OR "accountable" OR "answerab*" OR "principl*" OR "justified" OR "justification" OR "pre-departure training" OR "predeparture training" OR "post-return" OR "postreturn" OR "practice guideline*"))) AND ((title:("International educational exchang*" OR "global health stud*" OR "global health elective*" OR "global health educat*" OR "global health program*" OR "global health intern*" OR "medical elective*" OR "medical volunteer*" OR "international apprenticeship*" OR "international health elective*" OR "international intern*" OR "international clinical clerkship*" OR "international clinical rotation*" OR "international clinical experience*" OR "international rotation*" OR "practical training" OR "short-term international trip*" OR "clinical clerkship*" OR "clinical elective*" OR "international medical traineeship*" OR "short-term experience*" OR "short-term international medical initiative*" OR "short-term medical initiative*" OR "short-term elective" OR "international medical initiative*" OR "international medical endeavor*" OR "global health educational opportunit*" OR "global health training*" OR "global health practicum*" OR "global health experience*" OR "global health outreach work" OR "service learning" OR "international clinical education*" OR "medical mission*" OR "global health mission*" OR "mission trip*" OR "bidirectional exchange*" OR "international cooperation" OR "residency education") OR ab:("International educational exchang*" OR "global health stud*" OR "global health elective*" OR "global health educat*" OR "global health program*" OR "global health intern*" OR "medical elective*" OR "medical volunteer*" OR "international apprenticeship*" OR "international health elective*" OR "international intern*" OR "international clinical clerkship*" OR "international clinical rotation*" OR "international clinical experience*" OR "international rotation*" OR "practical training" OR "short-term international trip*" OR "clinical clerkship*" OR "clinical elective*" OR "international medical traineeship*" OR "short-term experience*" OR "short-term international medical initiative*" OR "short-term medical initiative*" OR "short-term elective" OR "international medical initiative*" OR "international medical endeavor*" OR "global health educational opportunit*" OR "global health training*" OR "global health practicum*" OR "global health experience*" OR "global health outreach work" OR "service learning" OR "international clinical education*" OR "medical mission*" OR "global health mission*" OR "mission trip*" OR "bidirectional exchange*" OR "international cooperation" OR "residency education"))) AND yr:[2018 TO 2023]
